# Integrative Analysis of Transcriptome-Wide Association Study and mRNA Expression Profiles Identified Candidate Genes and Pathways Associated With Acute Myocardial Infarction

**DOI:** 10.3389/fgene.2021.616492

**Published:** 2021-02-02

**Authors:** Guanzhong Chen, Liwei Liu, Huanqiang Li, Zhubin Lun, Ziling Mai, Wenguang Lai, Enzhao Chen, Chunyun Zhou, Sijia Yu, Junqing Yang, Shiqun Chen, Jiyan Chen, Yong Liu

**Affiliations:** ^1^Guangdong Provincial Key Laboratory of Coronary Heart Disease Prevention, Department of Cardiology, Guangdong Provincial People’s Hospital, Guangdong Cardiovascular Institute, Guangdong Academy of Medical Sciences, Guangzhou, China; ^2^Guangdong Provincial People’s Hospital, School of Medicine, South China University of Technology, Guangzhou, China; ^3^The Second School of Clinical Medicine, Southern Medical University, Guangzhou, China; ^4^The First School of Clinical Medicine, Guangdong Medical University, Zhanjiang, China; ^5^Guangdong Provincial People’s Hospital, School of Biology and Biological Engineering, South China University of Technology, Guangzhou, China

**Keywords:** acute myocardial infarction, transcriptome-wide association study, genome-wide association study, mRNA expression profiles, biological process

## Abstract

**Background:**

Acute myocardial infarction (AMI), characterized by an event of myocardial necrosis, is a common cardiac emergency worldwide. However, the genetic mechanisms of AMI remain largely elusive.

**Methods:**

A genome-wide association study dataset of AMI was obtained from the CARDIoGRAMplusC4D project. A transcriptome-wide association study (TWAS) was conducted using the FUSION tool with gene expression references of the left ventricle and whole blood. Significant genes detected by TWAS were subjected to Gene Ontology (GO) enrichment analysis. Then the TWAS results of AMI were integrated with mRNA expression profiling to identify common genes and biological processes. Finally, the identified common genes were validated by RT-qPCR analysis.

**Results:**

TWAS identified 1,050 genes for the left ventricle and 1,079 genes for whole blood. Upon comparison with the mRNA expression profile, 4 common genes were detected, including HP (P_TWAS_ = 1.22 × 10^–3^, P_GEO_ = 4.98 × 10^–2^); CAMP (P_TWAS_ = 2.48 × 10^–2^, P_GEO_ = 2.36 × 10^–5^); TNFAIP6 (P_TWAS_ = 1.90 × 10^–2^, P_GEO_ = 3.46 × 10^–2^); and ARG1 (P_TWAS_ = 8.35 × 10^–3^, P_GEO_ = 4.93 × 10^–2^). Functional enrichment analysis of the genes identified by TWAS detected multiple AMI-associated biological processes, including autophagy of mitochondrion (GO: 0000422) and mitochondrion disassembly (GO: 0061726).

**Conclusion:**

This integrative study of TWAS and mRNA expression profiling identified multiple candidate genes and biological processes for AMI. Our results may provide a fundamental clue for understanding the genetic mechanisms of AMI.

## Introduction

Acute myocardial infarction (AMI), defined by an event of myocardial necrosis caused by the rupture of an atherosclerotic plaque, has become the leading cause of death and disability worldwide (Murray et al., 2015). Previous studies have identified several factors associated with the onset of AMI, including age, gender, hypertension, diabetes mellitus, smoking, alcohol consumption and physical exertion (Mittleman et al., 1993, 1995, 1999; Lipovetzky et al., 2007). Moreover, increasing evidence has demonstrated that genetic factors also contribute to the development of AMI. Genome-wide association studies (GWAS) have identified several genetic loci that confer susceptibility to AMI, such as CDKN2A/2B, CELSR2-PSRC1-SORT1, CXCL12, ABO, LDLR, and APOA5 (Qi et al., 2011; Do et al., 2015; Nikpay et al., 2015).

Although GWAS have successfully identified great numbers of loci, only a small proportion (approximately 10.6%) of phenotypic variance explains coronary artery disease or AMI (Deloukas et al., 2013). This is probably because significant single nucleotide polymorphisms (SNPs) identified by GWAS are mainly enriched in non-coding regulatory regions. Due to limited genomic annotation, it is difficult to explain the relative risk of diseases. Transcriptome-wide association study (TWAS) has identified significant gene expression-trait associations by integrating gene expression data with GWAS data (Gusev et al., 2016). Multiple studies have indicated that TWAS exhibited good performance in identifying novel genes of complex cardiovascular diseases, such as atrial fibrillation (Zhang et al., 2019) and calcific aortic valve stenosis (Thériault et al., 2018).

In this study, we conducted an integrative analysis of TWAS and mRNA expression profiles of AMI. TWAS was first performed on a large-scale GWAS from the CARDIoGRAMplusC4D Consortium (Nikpay et al., 2015) to identify novel associated genes. The AMI-associated genes identified by TWAS were further subjected to Gene Ontology (GO) analysis to explore the core biological processes or signaling pathways. TWAS results were finally compared to those of mRNA expression profiling to identify common genes.

## Materials and Methods

### GWAS Summary Datasets of AMI

We collected a large-scale GWAS summary data of coronary artery diseases from CARDIoGRAMplusC4D. Briefly, the study is a meta-analysis of 48 coronary artery disease studies. We chose one of the subgroup results (GWAS summary data for AMI, AMI patients, *n* = 43,676; controls, *n* = 1,28,199) to perform further TWAS analysis (Nikpay et al., 2015; Luo et al., 2019). SNP genotyping was conducted using a combination of array platforms, including Affymetrix GeneChip Human Mapping 500K, Affymetrix Genome-Wide Human SNP Array 6.0 and Illumina HumanHap300 BeadChip. In this dataset, an additive model where the log (genotype risk ratio) for a genotype was proportional to the number of risk alleles was used. For detailed information on the cohorts, genotyping, and quality control approaches, please refer to the published studies.

### mRNA Expression Profiles of AMI

The mRNA expression profiles of AMI were obtained from two series of GEO datasets (GEO accession ID: GSE29532 and GSE61144). These two datasets were generated using Affymetrix Human Exon 1.0 ST Array (GPL5175) and Sentrix Human-6 v2 Expression BeadChip (GPL6106), respectively. For detailed information, please refer to original publications (Silbiger et al., 2013; Park et al., 2015). We used the online tool GEO2R to conduct differential gene expression analysis comparing the expression of whole blood mRNA between AMI cases and controls. Genes with fold change (FC) ≥2 or ≤0.5 and *P* < 0.05 were defined as differentially expressed genes (DEGs).

### TWAS Analysis

TWAS analysis of AMI was performed using FUSION software. Briefly, FUSION used a relatively small set of individuals with both genotype and gene expression data to compute gene expression weights for different tissues. TWAS analysis uses pre-computed gene expression weights together with GWAS summary statistics to calculate the association of every gene to a given disease. The genetic values of expression are computed one probe set at a time using SNP genotyping data located 500 kb on either side of the gene boundary. In this study, gene expression weights of whole blood and left ventricle were derived from the FUSION website^1^, which is attached to the GTEx database. Genes with significant and suggestive association signals were identified by *P* < 0.05.

### Functional Characterization of GO and KEGG Analysis

Associated genes identified by TWAS and mRNA expression profiles were subjected to GO and KEGG analysis. Analyses were evaluated using the Database for Annotation, Visualization and Integrated Discovery (DAVID) bioinformatics tool^2^. A GO term or KEGG pathway with adjusted *P*-value < 0.05 was considered statistically significant (Huang da et al., 2009). Visualization of GO analysis was conducted with R package “ggplot2.”

### Animals

Adult male Sprague-Dawley (SD) rats weighing 200–220 g were purchased from the Experimental Animal Center at Guangzhou University of Chinese Medicine (Guangzhou, China). The animal facilities and protocols were approved by the Guangdong provincial people’s hospital Ethics Committee. Before the beginning of the experiments, all animals were allowed to acclimate to the facility in a standard SPF-class animal room for 1 week. Six rats were randomly divided into sham and cardiac ischemia and reperfusion groups, each group consisting of three rats. Before experiments, rats were fasted overnight and deprived of water.

### Acute Myocardial Infarction Rat Model Establishment

Rats were weighed and anesthetized with 10% chloral hydrate (0.4 ml/100 g) by intraperitoneal injection. Tracheostomy was performed and rats were ventilated with room air from a positive pressure rodent ventilator. Lead II electrocardiography was recorded throughout the experiment. The left anterior descending coronary artery was then identified and ligated at approximately 2 mm distal to its origin after a left-side thoracotomy at the fourth intercostal space and heart exposure. A small vinyl tube was used to occlude the LAD by pulling the thread. A ligature for 30 min was then followed by a 24 h reperfusion in the AMI group. The same experimental performance was conduct in the sham group except occluding the LAD. ST-elevation from electrocardiography was used to confirm the establishment of the AMI rat model. At the end of the study, the animals were sacrificed and the hearts were collected for subsequent experiments.

### Hematoxylin-Eosin (HE) Staining

Rat heart tissues were fixed in 10% neutral buffered formalin for more than 24 h and then embedded in paraffin. Tissue sections were cut as 4 mm-thick using a microtome. Histopathological evaluation of the tissue sections was conducted by hematoxylin-eosin staining. Finally, tissue morphology was observed.

### Reverse Transcription Quantitative Real-Time Polymerase Chain Reaction (RT-qPCR) Analysis

RT-qPCR was used to verify the expression of the identified genes in heart tissues of both AMI and sham rats. Total RNA was extracted from the collected heart from the AMI rats and sham rats by Trizol Universal reagent (TIANGEN, DP424). Reverse transcription was then carried out using total RNA. cDNA was synthesized via reverse transcription using a PrimeScript^TM^ RT Reagent Kit (RR037) according to the manufacturer’s protocols (Takara, Japan). Then, quantitative real-time PCR was carried out using TB Green^®^ Premix Ex Taq^TM^ II (Tli RNaseH Plus, RR820A) according to the manufacturer’s protocols (Takara, Japan). The primers used to amplify genes in the reactions were synthesized by Generay (Shanghai, China). All RT-qPCR primer sequences are shown in Supplementary Table S4. qPCR was performed in a CFX Connect Real-Time System (Bio-Rad, United States). The relative expression levels of the miRNAs were normalized to those of the reference gene GAPDH and calculated using the 2^–▲▲CT^ method. All reactions were repeated in triplicate.

## Results

### TWAS Analysis Results

A total of 1,862 genes were identified by TWAS across the two tissues (Supplementary Tables S1, S2). For the left ventricle, TWAS identified 1,050 genes with TWAS *P* < 0.05, such as SURF2 (P_TWAS_ = 1.25 × 10^–12^), RP11-378J18.8 (P_TWAS_ = 8.33 × 10^–11^), ABO (P_TWAS_ = 1.70 × 10^–7^), ICA1L (P_TWAS_ = 1.78 × 10^–7^), and MIA3 (P_TWAS_ = 2.33 × 10^–7^). For whole blood, TWAS identified 1,079 genes with TWAS *P* < 0.05, such as PSRC1 (P_TWAS_ = 1.18 × 10^–10^), CARF (P_TWAS_ = 2.41 × 10^–10^), RP11-378J18.8 (P_TWAS_ = 4.83 × 10^–9^), GGCX (P_TWAS_ = 1.01 × 10^–8^), and POC1B (P_TWAS_ = 1.07 × 10^–8^). Among all these genes, 266 were detected in both the left ventricle and whole blood. The top 20 AMI associated genes identified by TWAS are shown in Table 1.

**TABLE 1:** List of top 20 candidate genes identified by TWAS for AMI.

**Gene**	**Chromosome**	**Best GWAS ID**	**TWAS *P*-value**	**Comparative tissue**
SURF2	9	rs495828	1.25 × 10^–12^	Heart left ventricle
RP11-378J18.8	1	rs17163358	8.33 × 10^–11^	Heart left ventricle
PSRC1	1	rs7528419	1.18 × 10^–10^	Whole blood
CARF	2	rs6722332	2.41 × 10^–10^	Whole blood
RP11-378J18.8	1	rs17163358	4.83 × 10^–9^	Whole blood
GGCX	2	rs1561198	1.01 × 10^–8^	Whole blood
POC1B	12	rs2681472	1.07 × 10^–8^	Whole blood
IL6R	1	rs4845618	7.09 × 10^–8^	Whole blood
SH2B3	12	rs653178	9.54 × 10^–8^	Whole blood
FAM177B	1	rs17163358	1.36 × 10^–7^	Whole blood
TAF1A	1	rs17163358	1.39 × 10^–7^	Whole blood
ABO	9	rs495828	1.70 × 10^–7^	Heart left ventricle
ICA1L	2	rs6722332	1.78 × 10^–7^	Heart left ventricle
MIA3	1	rs17163358	2.33 × 10^–7^	Heart left ventricle
BSND	1	rs11591147	2.52 × 10^–7^	Whole blood
KIAA1462	10	rs2505083	2.60 × 10^–7^	Heart left ventricle
UBE2Q1	1	rs4845618	5.30 × 10^–7^	Whole blood
RP11-422P24.10	1	rs4845618	6.23 × 10^–7^	Whole blood
CDKN2A	9	rs4977574	7.47 × 10^–7^	Whole blood
SRD5A3-AS1	4	rs11945371	9.05 × 10^–7^	Whole blood
				

### Integrative Analysis of TWAS and mRNA Expression Profiles

After further comparison of differentially expressed genes identified by TWAS and mRNA expression profiles, we identified four overlapping genes, including HP (P_TWAS_ = 1.22 × 10^–3^, P_GEO_ = 4.98 × 10^–2^); CAMP (P_TWAS_ = 2.48 × 10^–2^, P_GEO_ = 2.36 × 10^–5^); TNFAIP6 (P_TWAS_ = 1.90 × 10^–2^, P_GEO_ = 3.46 × 10^–2^); and ARG1 (P_TWAS_ = 8.35 × 10^–3^, P_GEO_ = 4.93 × 10^–2^). Detailed information on the common genes is shown in Table 2.

**TABLE 2:** List of overlapping candidate genes identified by TWAS and GEO mRNA profiling for AMI.

**Gene**	**Chromosome**	**GEO *P*-value**	**TWAS *P*-value**	**Comparative tissue**
HP	16	4.98 × 10^–2^	1.22 × 10^–3^	Heart left ventricle
CAMP	3	2.36 × 10^–5^	2.48 × 10^–2^	Heart left ventricle
TNFAIP6	2	3.46 × 10^–2^	1.90 × 10^–2^	Whole blood
ARG1	6	4.93 × 10^–2^	8.35 × 10^–3^	Whole blood

### Functional Analyses

**FIGURE 1:**
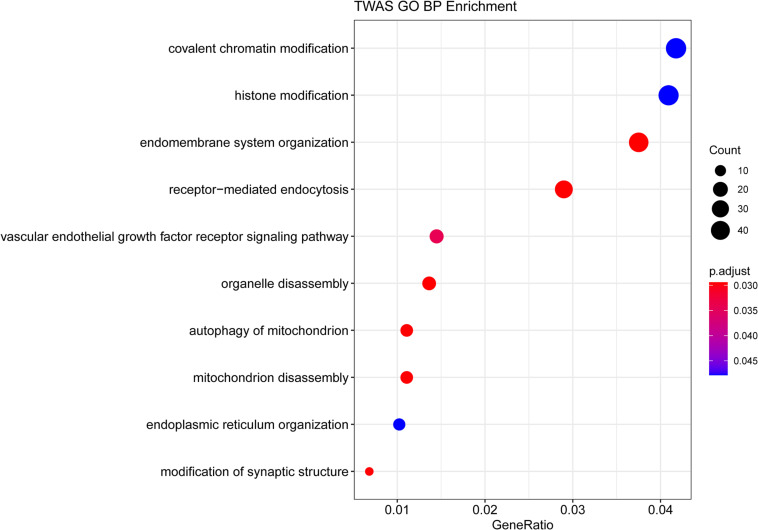
GO enrichment analysis of the genes identified by TWAS.

GO enrichment analysis of genes identified by TWAS detected 10 significant biological process terms, such as organelle disassembly (GO: 1903008), modification of synaptic structure (GO: 0099563), autophagy of mitochondrion (GO: 0000422) and mitochondrion disassembly (GO: 0061726). The associated GO terms identified by TWAS are listed in Figure 1 and Table 3. For KEGG pathway enrichment analysis (Supplementary Table S3), 5 significant pathways were detected, including bacterial invasion of epithelial cells (hsa05100, adjust *P*-value = 1.30 × 10^–2^), insulin resistance (hsa04931, adjust *P*-value = 1.30 × 10^–2^), proteoglycans in cancer (hsa05205, adjust *P*-value = 1.73 × 10^–2^), focal adhesion (hsa04510, adjust *P*-value = 4.80 × 10^–2^) and regulation of actin cytoskeleton (hsa04810, adjust *P*-value = 4.87 × 10^–2^).

**TABLE 3:** Gene ontology enrichment analysis results of AMI associated genes identified by TWAS.

**GO ID**	**Description**	**Adjust *P*-value**	**Gene count**
GO:0016569	Covalent chromatin modification	4.78 × 10^–2^	49
GO:0016570	Histone modification	4.78 × 10^–2^	48
GO:0010256	Endomembrane system organization	2.94 × 10^–2^	44
GO:0006898	Receptor-mediated endocytosis	2.94 × 10^–2^	34
GO:0048010	Vascular endothelial growth factor receptor signaling pathway	3.43 × 10^–2^	17
GO:1903008	Organelle disassembly	2.94 × 10^–2^	16
GO:0000422	Autophagy of mitochondrion	2.94 × 10^–2^	13
GO:0061726	Mitochondrion disassembly	2.94 × 10^–2^	13
GO:0007029	Endoplasmic reticulum organization	4.78 × 10^–2^	12
GO:0099563	Modification of synaptic structure	2.94 × 10^–2^	8

### qPCR Validation

**FIGURE 2:**
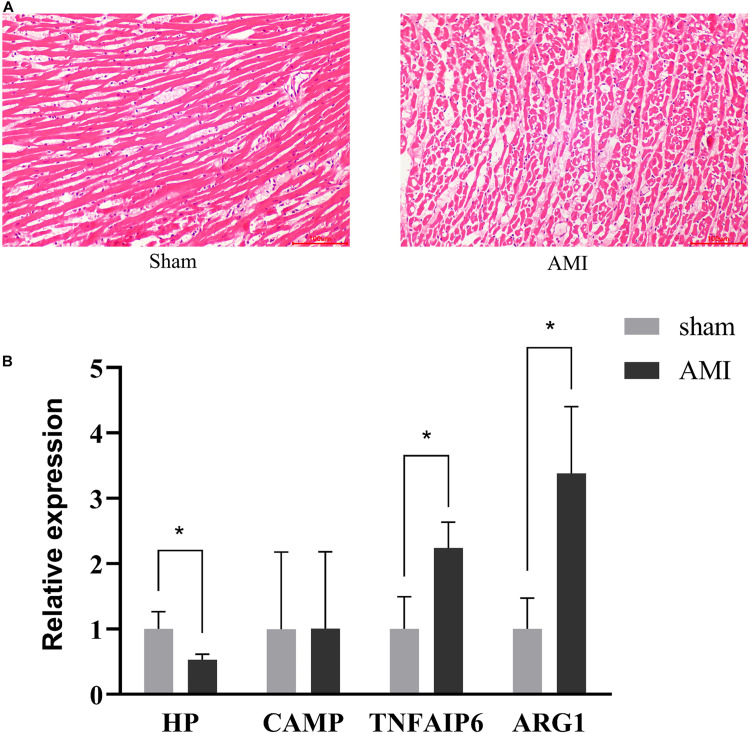
Validation of identified gene expression. **(A)** Pathological changes in rat myocardium tissues (HE staining × 200). **(B)** RT-qPCR analysis of four identified genes. Rat GAPDH was used as internal controls. The measurement data are expressed as the means ± *SD*. *P < 0.05.

To confirm myocardial infarction, HE staining was performed to detect the histopathological changes between the AMI group and sham group. Comparing with the normal cardiac histology in the sham group, the AMI group showed significant inflammatory cells infiltration, cardiomyocyte necrosis and fiber disruption (Figure 2A). RT-qPCR was used to confirm the expression of the identified genes in sham and AMI rat hearts. As shown in Figure 2B, the HP was significantly down-regulated, while TNFAIP6 and ARG1 were significantly up-regulated. However, the expression of CAMP was not significant. In general, RT-qPCR results confirmed the integrative analysis result of TWAS and mRNA profiles.

## Discussion

To demonstrate the implication and potential effects of genetic factors in the development of AMI, we performed TWAS by using large-scale GWAS data. Then, we compared the TWAS results with the mRNA expression profiles to explore common genes, in which 4 genes overlapped, including HP, CAMP, TNFAIP6, and ARG1. GO enrichment suggested that 10 biological processes, including autophagy of mitochondrion (GO: 0000422) and mitochondrion disassembly (GO: 0061726) were associated with AMI.

In our study, one of the notable genes identified was CAMP. CAMP encodes a member of the antimicrobial peptide family, characterized by a highly conserved N-terminal signal peptide containing a cathelin domain and a structurally variable cationic antimicrobial peptide (Zasloff, 2002; Hilchie et al., 2013). In addition to its antibacterial activities, the CAMP protein functions in cellular chemotaxis and inflammatory response regulation (Lande et al., 2007). Previous studies indicated that CAMP, acting as an activator of Akt, ERK and FoxO3a, protects against cardiomyocyte apoptosis in AMI (Bei et al., 2019). Moreover, Bei et al. (2019) showed that serum levels of LL-37 (human analog of CAMP) were significantly reduced in AMI patients. Low-level LL-37 might also be associated with a worse prognosis in AMI. Furthermore, other studies showed that CAMP was related to dyslipidemia (Chamorro et al., 2009; Dashty et al., 2014), indicating that CAMP might induce lipid abnormalities to increase the risk of AMI.

Another susceptibility gene was TNFIP6, also called TSG-6. TNFIP6 encodes a secretory protein of the hyaluronan-binding protein family, which is important in the protease network associated with inflammation (Milner et al., 2006; Heng et al., 2008). Accumulating evidence supports that inflammation plays an important role in the development of AMI (Wisniewski and Vilcek, 2004; Milner et al., 2006). A previous study showed that human multipotent stromal cells (hMSCs) enhance the repair of myocardial tissue during AMI. After knockdown of TSG-6 in hMSCs, positive improvements in inflammatory responses, infarct size, and cardiac function induced by hMSC injection were significantly attenuated (Lee et al., 2009). Therefore, TNFIP6 may represent an important gene involved in the development of AMI by suppressing the inflammatory response.

ARG1, also called arginase 1, was another gene identified by TWAS analysis. It was first studied in the urea cycle (Iyer et al., 1998), which can catalyze the hydrolysis of arginine and then convert arginine to ornithine and urea (Sin et al., 2015). However, ARG1 also could induce endothelial dysfunction and promote the progress of atherosclerosis by competing with nitric oxide synthase (NOS) for the common substrate-L-arginine and inhibited of biosynthesis of nitric oxide (NO) (Santhanam et al., 2007). It is these kinds of functions making ARG1 possible to participate in the development of AMI. Several studies have revealed this relationship between ARG1 and AMI. Zhu et al. (2015) found out that ARG1 can downregulate NO biosynthesis and promote the enrichment of inflammatory cells into infarcted heart areas in mice. In the latest research (Zhang et al., 2020), ARG1 was determined as a potential biomarker for AMI with the area under the receiver operating characteristic curve (AUC) up to 0.776. All together the application value of ARG1 in AMI is worth expecting.

The haptoglobin (Hp) gene locus at chromosome 16q22 is polymorphic with two alleles (Bowman and Kurosky, 1982). Interestingly, it is reported that Haptoglobin phenotypes differ in their ability in different diseases (Melamed-Frank et al., 2001; Bamm et al., 2004; Asleh et al., 2005). This gene has been revealed to play an important role in AMI. Hp 1-1 phenotype was determined to have an association with increased risk of AMI incidence in Chinese patients with T2D in the latest research (Gurung et al., 2019). Suleiman et al. (2005) have reported that DM individuals with the Hp 2 allele have significantly larger myocardial infarctions than DM individuals homozygous for the 1 allele, which was confirmed in mice by Blum et al. (2007). The underlying mechanism of this relationship may be due to increased oxidative stress in DM Hp 2 mice (Blum et al., 2007). Therefore, Hp is also an important susceptibility gene in the development and prognosis of AMI.

Functional enrichment analysis identified mitochondrial function related GO terms (GO: 0000422 and GO: 0061726) were enriched. It is well known that mitochondria are a source of energy for cardiomyocyte cells since they produce most of the adenosine triphosphate. Mitochondrial dysfunction at the onset of acute myocardial ischemia is a critical determinant of cell death in AMI patients (Hernandez-Resendiz et al., 2020). Also, efficient mitophagy response may allow cardiomyocytes to alleviate the hypoxia or nutritional stress (Lopez-Crisosto et al., 2017). Kubli et al. (2013) found that Parkin-deficient mice had significantly reduced mitophagy and accumulated dysfunctional mitochondria after myocardial infarction, leading to exacerbated cardiac injury and reduced survival. Along similar lines, pre- or post-conditioning could also mediate cardio-protective effects by activating the mitophagy during AMI (Huang et al., 2011; Wu et al., 2013; Yu et al., 2015). Additionally, a previous study showed that inflammatory conditions or arginine catabolism could affect mitochondrial function (Lee and Huttemann, 2014; Tang et al., 2020). Sun et al. indicated that the anti-inflammatory effects of CAMP were dependent its ability to stimulate mitochondrial biogenesis and maintain mitochondrial homeostasis (Sun et al., 2014). Tang et al. (2020) also found that the loss of ARG1 could cause arginine accumulation and mitochondrial dynamics disruption. Taken all together, the common genes shared by TWAS analysis and mRNA profiles may play key roles in the development of AMI by mediating the mitochondrial dysfunction.

To the best of our knowledge, this is the first large-scale comprehensive study in AMI combining TWAS and mRNA gene expression profiles. By integrating the GWAS data with gene expression profiles, common genes and potential pathways might be more convincing. However, some limitations of this study need to be noted. First, although TWAS was conducted to identify several casual genes associated with AMI, other loci that are independent of cis-gene expression effects may have been missed. Second, the novel candidate genes and pathways related to AMI resulted from bioinformatics analysis. Further functional studies are needed to confirm our findings and to clarify the potential biological processes underlying AMI.

## Conclusion

In conclusion, we integrated a tissue-related TWAS and gene expression profile to identify 4 common genes and biological pathways related to AMI. These results may provide novel insights into investigating the pathogenesis of AMI and serve as a fundamental clue for understanding the genetic mechanisms of AMI.

## Data Availability Statement

The datasets presented in this study can be found in online repositories. The names of the repository/repositories and accession number(s) can be found in the article/Supplementary Material.

## Ethics Statement

The animal study was reviewed and approved by the Guangdong provincial people’s hospital Ethics Committee.

## Author Contributions

GC, LL, YL, and JC: conception and design. HL, ZL, and SC: administrative support. GC, HL, and ZM: collection and assembly of data. GC and SC: data analysis and interpretation. All authors: wrote and approved the final manuscript.

## Conflict of Interest

The authors declare that the research was conducted in the absence of any commercial or financial relationships that could be construed as a potential conflict of interest.

## Supplementary Material

The Supplementary Material for this article can be found online at: https://www.frontiersin.org/articles/10.3389/fgene.2021.616492/full#supplementary-material

## Footnotes

1 https://gusevlab.org/projects/fusion/

2 https://david.ncifcrf.gov/
